# Conflict Resolution as Near-Threshold Decision-Making: A Spiking Neural Circuit Model with Two-Stage Competition for Antisaccadic Task

**DOI:** 10.1371/journal.pcbi.1005081

**Published:** 2016-08-23

**Authors:** Chung-Chuan Lo, Xiao-Jing Wang

**Affiliations:** 1 Institute of Systems Neuroscience, National Tsing Hua University, Hsinchu, Taiwan; 2 Center for Neural Science, New York University, New York, New York, United States of America; 3 NYU-ECNU Institute of Brain and Cognitive Science at NYU Shanghai, Shanghai, China; University College London, UNITED KINGDOM

## Abstract

Automatic responses enable us to react quickly and effortlessly, but they often need to be inhibited so that an alternative, voluntary action can take place. To investigate the brain mechanism of controlled behavior, we investigated a biologically-based network model of spiking neurons for inhibitory control. In contrast to a simple race between pro- versus anti-response, our model incorporates a sensorimotor remapping module, and an action-selection module endowed with a “Stop” process through tonic inhibition. Both are under the modulation of rule-dependent control. We tested the model by applying it to the well known antisaccade task in which one must suppress the urge to look toward a visual target that suddenly appears, and shift the gaze diametrically away from the target instead. We found that the two-stage competition is crucial for reproducing the complex behavior and neuronal activity observed in the antisaccade task across multiple brain regions. Notably, our model demonstrates two types of errors: fast and slow. Fast errors result from failing to inhibit the quick automatic responses and therefore exhibit very short response times. Slow errors, in contrast, are due to incorrect decisions in the remapping process and exhibit long response times comparable to those of correct antisaccade responses. The model thus reveals a circuit mechanism for the empirically observed slow errors and broad distributions of erroneous response times in antisaccade. Our work suggests that selecting between competing automatic and voluntary actions in behavioral control can be understood in terms of near-threshold decision-making, sharing a common recurrent (attractor) neural circuit mechanism with discrimination in perception.

## Author Summary

We propose a novel neural circuit mechanism and construct a spiking neural network model for resolving conflict between an automatic response and a volitional one. In this mechanism the two types of responses compete against each other under the modulation of top-down control via multiple neural pathways. The model is able to reproduce a wide range of neuronal and behavioral features observed in various studies and provides insights into not just how subjects make correct responses and fast errors, but also why they make slow errors, a type of error often overlooked by previous modeling studies. The model suggests critical roles of tonic (non-racing) top-down inhibition and near-threshold decision-making in neural competition.

## Introduction

A hallmark of behavioral flexibility is our ability, given the same sensory input, to resolve the conflict between an automatic response and a more appropriate volitional one [1–3]. This ability requires at least two executive processes. First, an automatic (habitual or reflexive) response needs to be withheld by top-down inhibitory control [2–11]. Second, a flexible mapping between the sensory input and motor response must be executed based on a rule signal [12–15].

In laboratory, competition between automatic and volitional responses is often investigated using the antisaccade task [16], where a visual target suddenly appears in the periphery and one is required to make a saccade in the opposite direction from the target, rather than toward it as a prepotent reflex. Antisaccade has been used in clinical studies for testing the development of executive control or for probing its abnormalities associated with attention deficit hyperactivity disorder (ADHD) and other neurological and psychiatric disorders [17–22]. In addition to dorsal lateral prefrontal cortex (DLPFC) [23–26], extensive studies have identified several other correlated brain regions, which include frontal eye field (FEF), supplementary eye field (SEF), superior colliculus (SC), anterior cingulate cortex (ACC), parietal cortex and basal ganglia (see Munoz & Everling 2004 [16] and Pierrot-Deseilligny et al. 2005 [27] for review). These studies revealed a diversity in neuronal activities within or across brain regions during antisaccadic eye movement, suggesting that they are accomplished through complex interactions between multiple brain regions.

**Fig 1:**
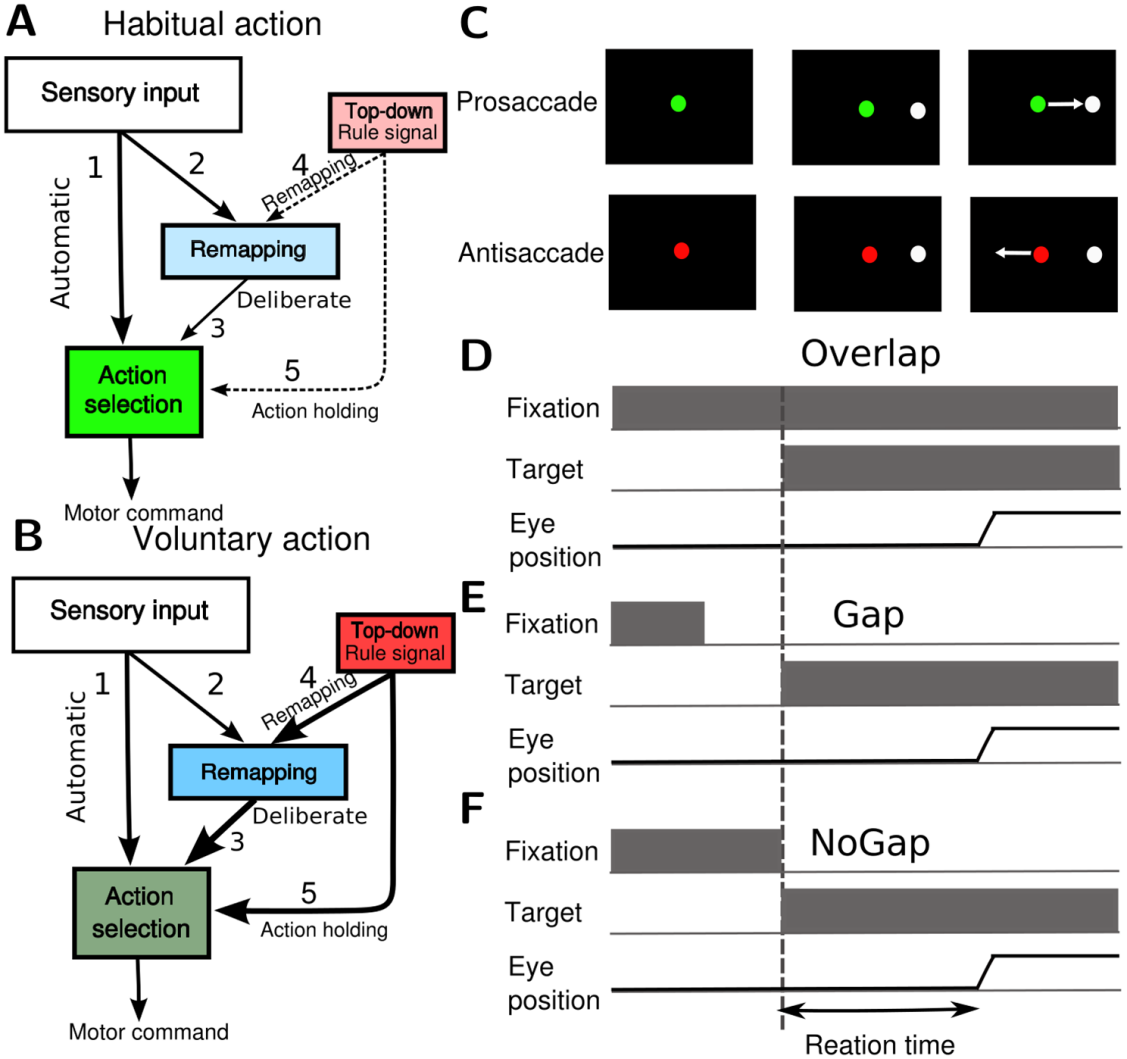
The hypothetical neural process of conflict resolution and the antisaccade tasks used in the study. Two main neural modules, the action selection and the remapping modules, are involved in the antisaccade process. **A.** At the onset of the sensory stimulus, the sensory signal drives the action selection module through the automatic pathway 1 and the remapping module through the deliberate pathways 2 & 3. When performing an automatic action driven by the stimulus, the subject does not need to apply a strong top-down control and the remapping module is in the baseline state which does not change the default sensorimotor mapping. **B**. In contrast, if the subject performs a voluntary action against the automatic one, a strong top-down control is required. The top-down control suppresses the automatic response by temporally inhibiting the action selection module through the pathway 5 and promotes the sensorimotor remapping by facilitating the remapping module through the pathway 4. **C** The antisaccade task. In the task the color of the fixation signal on the center of the screen serves as the cue for the trial type. In prosaccade trials (top), the subject has to make a saccadic eye movement toward the white target as soon as it appears. In antisaccade trials (bottom), the subject has to make a saccade away from the target. In the present study we simulated three types of antisaccade tasks: Overlap, Gap and NoGap. **D.** In the Overlap paradigm, the fixation signal stays on throughout the entire trial. **E.** In the Gap paradigm, the fixation signal is turned off 200 ms before the onset of the target. **F.** In the NoGap paradigm, the fixation signal is turned off at the same time with the onset of the target.

Despite extensive studies, the neural circuit mechanism underlying antisaccade remains poorly understood. A number of computational models have been proposed to reproduce some of the basic features observed in antisaccade or similar anti-reach movements [28–34]. Because the neural processes required by these movements were thought to be comparable to those proposed for Stroop tasks, most models are conceptually similar to the classic model in Cohen et al. 1990 [1] (see also Miller & Cohen 2001 [2]). These models typically assume a competition between a fast automatic response pathway and a slower voluntary response pathway, and a top-down control signal dictates which one wins the competition. An error (a saccade toward rather than away from the target) is attributed to a failure to suppress the automatic response, therefore is inevitably associated with a short reaction time. However, experiments with the antisaccade task have shown diversity in erroneous responses; there are fast errors associated with express saccade, but slow errors with reaction times comparable to those of correct antisaccade were also observed. In this work, we propose that competition takes place both at the level of interplay between automatic and voluntary responses, and within a neural circuit module for flexible sensorimotor remapping (Fig 1A and 1B). We implemented this mechanism in a biophysically realistic model of spiking neurons, using a previously proposed decision-making circuit [35, 36] and an inhibitory control circuit [37, 38]. We show that the model reproduces salient neurophysiological observations of diverse neural activity patterns (so far unaccounted for by previous simpler and more abstract models), as well as broad reaction time distributions for erroneous responses in antisaccade trials. This work revealed a new component of inhibitory control process (responsible for slow behavioral errors) which share a similar recurrent (attractor) neural circuit mechanism as that for near-threshold discrimination in perception [35, 39].

## Results

### Two-stage competitions

**Fig 2:**
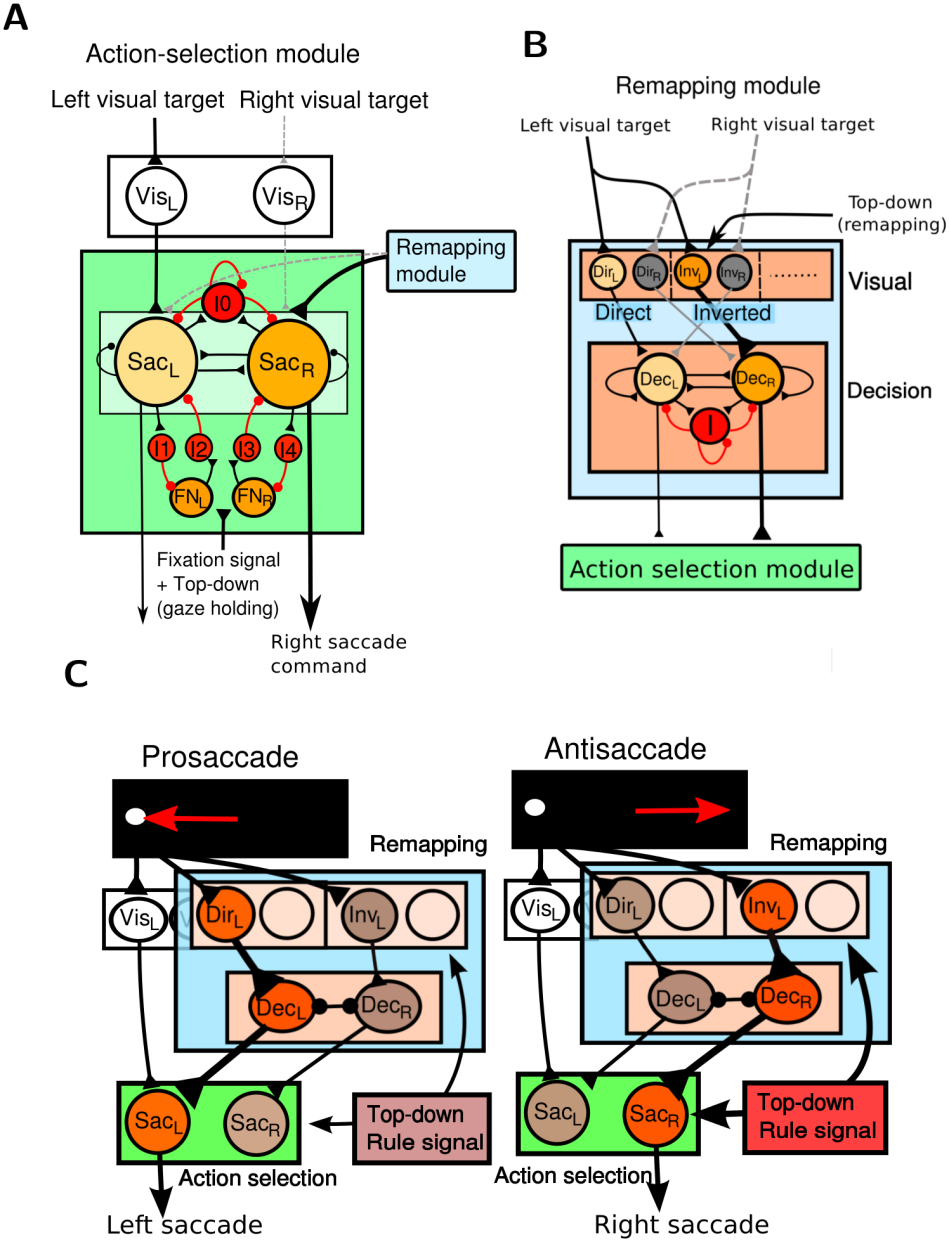
The detailed model circuits for antisaccade task. **A.** The action-selection module. The two excitatory populations of saccade neurons (Sac_*L*_ & Sac_*R*_) receive sensory input and modulation from the remapping module. The two populations compete with each other through the inhibitory interneuron population (I_0_) and also form mutual inhibitions with the fixation neuron populations FN_*L*_ or FN_*R*_ through inhibitory interneuron populations I_1_-I_4_. FN_*L*_ and FN_*R*_ are driven by the fixation signal and the top-down gaze-holding control which depends on the rule signal. **B.** The remapping module. The visual layer in the remapping module consists of neural populations that represent all possible sensorimotor maps including the direct map (the strongest by default) and the inverted map, which can be facilitated after training. The signals from the direct and inverted maps compete with each other in the decision layer and the outcome drives the downstream action-selection module. **C.** Schematics of anti- and prosaccade responses. In a prosaccade trial, the left visual signal directly triggers a left saccade by activating Sac_*L*_. The signal from the direct map in the remapping module may also contribute to the generation of saccades. During an antisaccade, the saccade neurons in Sac_*L*_ and Sac_*R*_ are temporally suppressed by the top-down gaze-holding control. In the mean time the top-down control facilitates the visual response of the inverted map, which strongly activates the decision population Dec_*R*_. As a result, Dec_*R*_ wins the competition and activates Sac_*R*_. Note that we neglect the decussation of the nervous systems in order to avoid complexity in the graphical representation.

In the proposed model, an antisaccadic eye movement was produced through competitions between the automatic response driven by the visual input and the voluntary response generated internally by the top-down controls. The competition occurred not just in one, but in multiple regions in the brain (Fig 1A and 1B). The action-selection module (Fig 2A), which produced the neuronal command that drove the eye movement, received the target signal directly from the visual neurons (Vis) and the signal representing the voluntary action from the remapping module (Fig 2B). The function of the remapping module is to convert the target signal into a desired saccade command based on the task instruction (pro- or antisaccade).

In a prosaccade trial, the remapping module was only weakly activated and the level of the gaze-holding control was low, so the target signal from the visual neurons Vis quickly triggered a prosaccade (Fig 2C left). In an antisaccade trial, the top-down controls took the effect by suppressing the action-selection module temporarily and activating the saccade command conversion in the remapping module. The target signal from the visual neurons Vis could not trigger an erroneous prosaccade due to the temporary suppression of the action-selection module. In the mean time the remapping module converted the target signal into a saccade command toward the end point that is opposite to the target. The remapping module took time to generate a decision signal and sent it to the action-selection module (Fig 2C right). By the time the suppression of the action-selection module from the top-down control was removed, the antisaccade signal was able to drive a correct antisaccade response.

### The action-selection module

The action-selection module chose between the signals that represented different actions and generated a corresponding motor command. We hypothesized that the function of action-selection module is performed, in part, in SC and FEF. Based on observations from various experiments on SC and FEF, we required the action-selection module to exhibit several basic functions: 1) The module needs to have two separate populations of saccade neurons. Each population triggers saccade eye movements to a different direction, i.e. right vs. left. A saccadic eye movement is defined as the population firing rate of one of the neural populations reaches a preset (100Hz) firing rate before the other does. 2) There is a strong competition between the two populations of saccade neurons, i.e. when one population is strongly activated, the other has to be suppressed. 3) Both populations of saccade neurons can be suppressed by fixation neurons when a gaze holding is required. To accommodate these functions, we created an action-selection module which consisted of the following neural populations (Fig 2A):

1) The excitatory populations Sac_*L*_ and Sac_*R*_ which signalled saccades to the left and right, respectively. 2) The inhibitory population I_0_ that provided mutual inhibition between Sac_*L*_ and Sac_*R*_. The circuit of Sac_*L*_, Sac_*R*_ and I_0_ was built based on a similar circuit design with that used in the decision layer [35]. The two saccade populations exhibited a winner-take-all competition and only one saccade population could be activated in each trial. 3) The excitatory populations FN_*L*_ and FN_*R*_ which contained fixation neurons for Sac_*L*_ and Sac_*R*_, respectively. 4) The inhibitory populations I_1_ -I_4_ which provided feedforward inhibition between the saccade neurons (Sac_*L*_ and Sac_*R*_) and fixation neurons (FN_*L*_ and FN_*R*_). The circuit formed by Sac_*L*_, Sac_*R*_, FN_*L*_, FN_*R*_ and I_1_-I_4_ was built based on the neural interactions reported in Munoz & Istvan (1998) [40]. Fixation neurons were driven by the fixation signal and gaze-holding signal from the top-down gaze-holding control.

Each of the saccade neural populations (Sac_*L*_ & Sac_*R*_) was further divided into two sub-populations, BN and BUN, which were used to mimic the observed burst neurons and build-up neurons, respectively [41]. The difference between BN and BUN was that BN neurons received strong feedback excitation by forming strong recurrent connections with all neurons in the Sac population while BUN received weaker feedback excitation by forming recurrent connections only with BN neurons (Table 1). When the input was weak, BUN neurons displayed a graded activity that resembled the observed build-up activity. When the input was large enough, BN neurons became activated and exhibited an all-or-none burst activity due to the strong recurrent excitation. Because there was mutual excitation between the two sub-populations, the burst activity in BN also strongly activated BUN neurons. As a result, both BUN and BN neurons exhibited strong burst activity before saccades as observed. In the paper, when we display the activity of the saccade neurons Sac, they are always represented by BUN neurons.

**Table 1:** Synaptic conductance (in nS) of connections in the action-selection module. Letters preceding each number indicate the type of receptor. N: NMDA, A: AMPA, G: GABA. Asterisks indicate the synapses that are endowed with short-term facilitation. See text for detail.

Source population (number of neurons)	Target population										
	Sac_*L*_		Sac_*R*_								
	BN_*L*_	BUN_*L*_	BN_*R*_	BUN_*R*_	I_0_	I_1_	I_2_	I_3_	I_4_	FN_*L*_	FN_*R*_
Vis_*L*_ (240)	A 0.05	A 0.05									
Vis_*R*_ (240)			A 0.05	A 0.05							
BN_*L*_ (250)	N 1.5	N 1.5			N 0.7*	N 0.7*					
BUN_*L*_ (80)	N 0.95				N 0.3*	N 0.7*					
BN_*R*_ (250)			N 1.5	N 1.5	N 0.7*			N 0.7*			
BUN_*R*_ (80)			N 0.95		N 0.3*			N 0.7*			
I_0_ (250)	G 1.0	G 1.0	G 1.0	G 1.0							
I_1_ (250)										G 0.7	
I_2_ (250)	G 0.3	G 0.1									
I_3_ (250)											G 0.7
I_4_ (250)			G 0.3	G 0.1							
FN_*L*_ (250)							N 0.15*				
FN_*R*_ (250)									N 0.15*		

Note that in the model BN neurons sent an efferent copy to neurons in the decision layer. The purpose of adding this projection was to shut down the neurons in the decision layer after an motor action was executed in order to generate a more realistic appearance of the neuronal activity in the decision layer. The projection did not affect the behavior performance or the conclusion of the present study.

### The remapping module

The remapping module consisted of two, visual and decision, layers (Fig 2B). The visual layer responded to the visual target and projected to the downstream decision layer. Empirical data suggest that the function of remapping module is, in part, performed in SEF and LIP [42, 46, 73–75]. We assumed that the projection from the remapping to the decision layers consisted of all possible visuomotor mappings that were capable of converting the target signal into a saccade command toward any end point. In the study we only modelled the two most relevant mappings: (i) the default direct map that directly transfers the target signal to a saccade command toward the target and (ii) the inverted map that converts the target signal into a saccade command toward the end point opposite to the target. The direct map was the strongest pathway by default, but the subject could learn to suppress it and to facilitate other maps based on the task instruction. The decision layer received inputs from these maps and made a probability decision on the stronger one through neural competition. The design of the decision layer followed that of the attractor neural network model of perceptual decision [35, 36]. The layer consisted of a large population of excitatory neurons and a population of inhibitory interneurons. Among the excitatory neurons, two sub-populations of neurons, Dec_*L*_ and Dec_*R*_, were selected to receive input from the visual layer and project to Sac_*L*_ and Sac_*R*_ in the action-selection module, respectively. Dec_*L*_ and Dec_*R*_ competed with each other through the inhibitory interneurons. Neurons are heavily connected to each other and form strong feedback excitations within each decision population (Dec_*L*_ or Dec_*R*_). There are weak excitatory connections between the two decision populations and the net interactions between them are inhibition due to the strong feedforward inhibition provided by the inhibitory interneurons (population *I*). The described circuit is consistent with the general organization of the cortical circuits in which the connection probability between pyramidal neurons decreases with the distance and the inhibitory interneurons provide feedback and feedforward inhibition to the pyramidal neurons.

**Fig 3:**
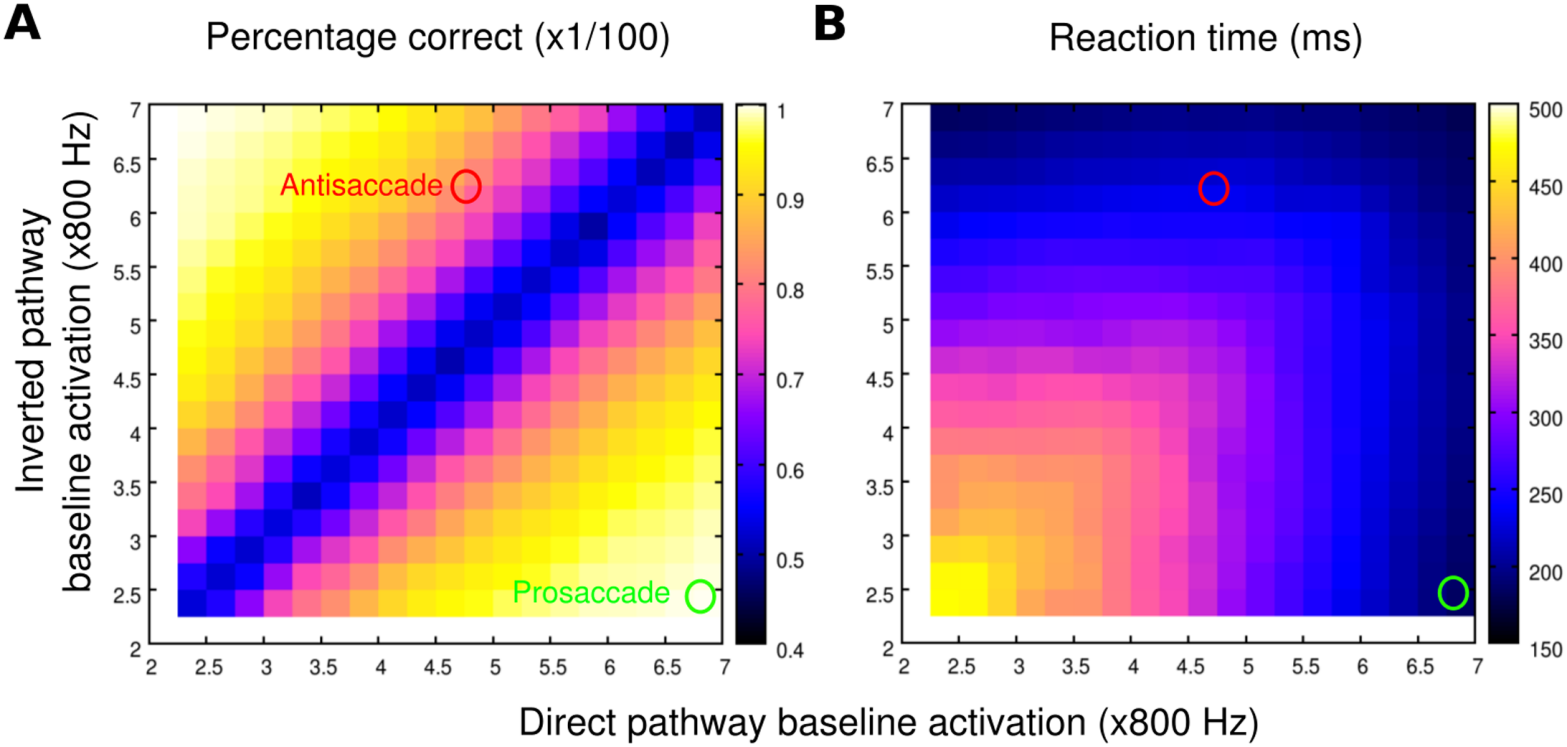
The reaction time and percentage correct associated with the decision layer. The decision layer is capable of producing the mean reaction time and the percentage correct observed in prosaccade and antisaccade tasks if the input levels are properly selected. We measured **A** the percentage of correct decisions and **B** the mean reaction times of the decision layer under various input levels from the upstream Dir and Inv neurons. Although the two types of neurons receive the same visual stimulus, their firing rates can be adjusted differently by varying their baseline inputs, which are indicated in the abscissa (for Dir) and in the ordinate (for Inv). The region above the diagonal line (where the input from the inverted pathway is stronger than that from the direct pathway) corresponds to the antisaccade condition and the region below corresponds to the prosaccade condition. A decision is counted as correct when the firing rate of the neural population driven by Dir or Inv hits a present decision threshold (50Hz) first in the prosaccade or antisaccade condition, respectively. The reaction time is the time interval between the onset of the input and the decision. We identified specific baseline input levels (green circles for prosaccades and red circles for antisaccades) which produce the mean reaction time and the percentage correct that match the typical values observed in monkey experiments. These specific baseline inputs were used to determined the strength of the top-down influence in the proposed model.

The neurons in the decision layer exhibited reverberatory excitation within each selective pool and winner-take-all competition between selective pools, which was essential in producing the observed longer mean reaction time and higher error rate in antisaccade trials than in prosaccade trials. The probability of making either choice and the response time depended on the levels of the inputs to Dec_*L*_ and Dec_*R*_ (Fig 3). Therefore, we first needed to individually decide the proper levels of inputs to the decision layer for prosaccades and antisaccades. The decision layer took inputs from Dir neurons in the direct map and Inv neurons in the inverted map. The neurons in both maps received the same visual signal but different baseline input, which was modulated by the top-down remapping control (see Materials and Methods). We tested the performance and the mean reaction time of the decision layer under various combinations of input levels by adjusting the background inputs to Dir and Inv neurons (Fig 3). Each combination of input was tested and averaged over 1000 trials. The optimal combination we found for prosaccade determined the default background input in the absence of the top-down control while the combination we found for antisaccade determined the levels of the background input, or *k*_Dir_ and *k*_Inv_, under the modulation of the top-down control.

### The role of the top-down control in antisaccade

We illustrate how the neural network model performs prosaccade and antisaccade using two example trials (Fig 2C). We first assume that the visual target appears on the left of the screen in both trials. In a prosaccade trial, the visual target activates left visual neurons in Vis_*L*_, Dir_*L*_ and Inv_*L*_. The activated Vis_*L*_ strongly excites Sac_*L*_ neurons. In the remapping module, Dir_*L*_ develops a stronger response than Inv_*L*_ does by default due to the stronger background excitation in Dir neurons. As a result, Dec_*L*_ wins the competition against Dec_*R*_ easily and sends the signal to Sac_*L*_. A leftward prosaccade is triggered by either the early signal from Vis_*L*_ or by the late signal from Dec_*L*_. In contrast, in an antisaccade trial, a subject suppresses the tendency to make a prosaccade by applying a strong top-down control which is assumed to be carried out, in part, by DLPFC (see Discussion). The top-down control causes two effects: 1) temporal suppression of Sac_*L*_ and Sac_*R*_ through the fixation neurons and 2) bias in the remapping module due to the facilitated inverted map and the suppressed direct map. See Materials and Methods for more detail. The left visual target, although strongly activates Vis_*L*_ as in the prosaccade trial, cannot activate Sac_*L*_ due to the suppression from the top-down holding control. In the remapping module, Inv_*L*_ sends a stronger signal to the decision layer than Dir_*L*_ does. In consequence, Dec_*R*_ wins the competition with probability higher than Dec_*L*_ does and triggers a rightward antisaccade by activating Sac_*R*_. Note that although the activity of Inv_*L*_ is stronger than that of Dir_*L*_, the difference in the magnitudes between the two populations is smaller than that in prosaccade trials. Therefore in antisaccade trials the activity of Dec_*R*_ ramps up slowly with a probability much less than 1. This produces a slower mean response time and a higher error rate in antisaccade than in prosaccade.

### Neuronal activity in prosaccade and antisaccade

**Fig 4:**
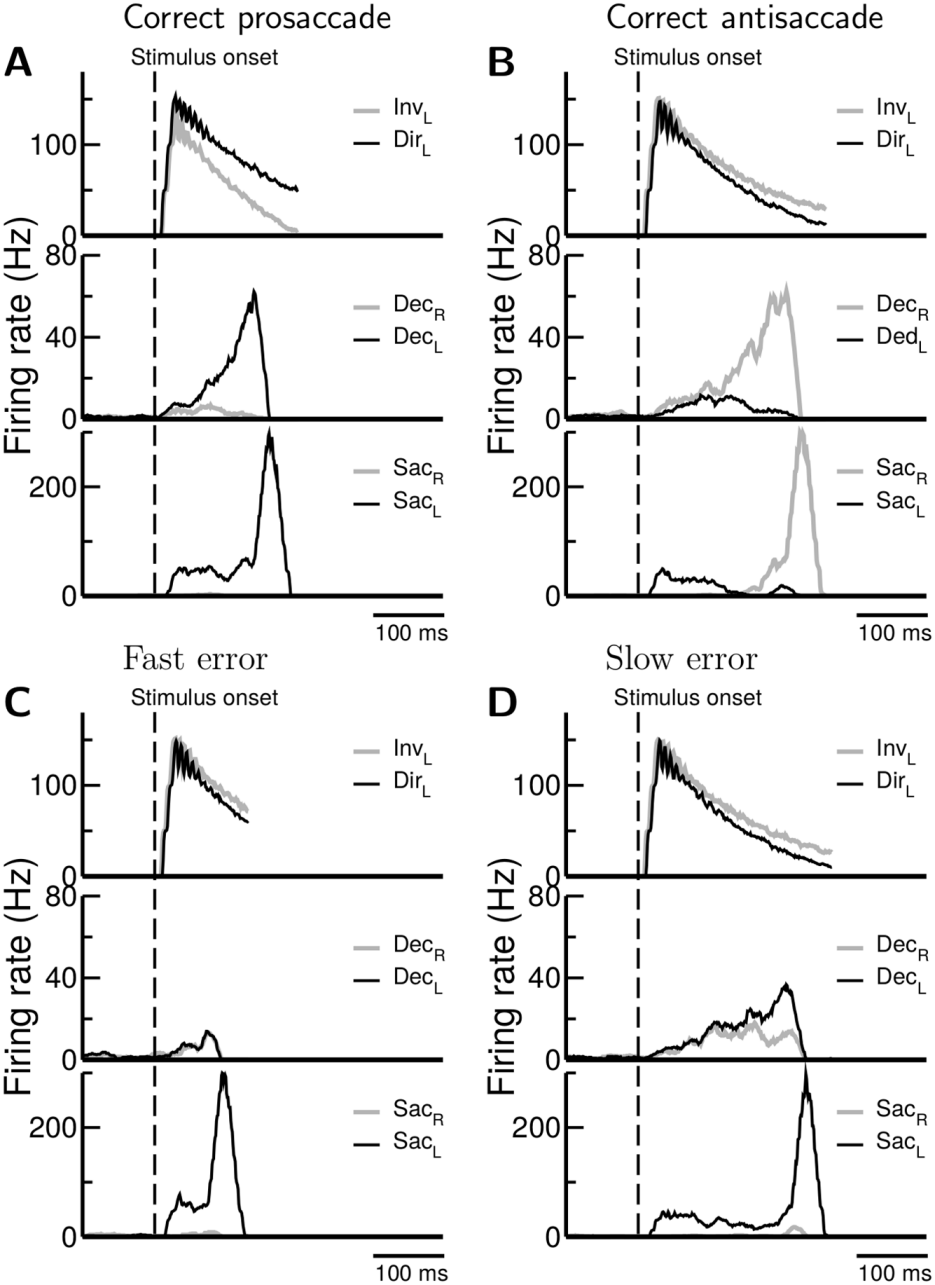
1 Simulated neural activity in prosaccade and antisaccade tasks. The model exhibits distinct neuronal activity (population firing rate) between prosaccade and antisaccade and between fast and slow errors, giving insight into how errors are produced. Here we display population firing rates from four sample trials in the Gap task and the visual target is on the left in all panels. Neural activity in the NoGap or Overlap tasks is qualitatively similar with that of the Gap task displayed here. **A.** Neurons in the direct map (Dir neurons) are dominant by default. Therefore they exhibit stronger response to the target signal in prosaccade trials. As a result, the corresponding downstream decision neurons (in Dec_*L*_) on the left win the competition against the decision neurons (in Dec_*R*_) on the right. Dec_*L*_ then activates the downstream saccade neurons (Sac_*L*_) and triggers a prosaccade. **B.** In antisaccade, the top-down control suppresses neurons in the direct map pathway (Dir_*L*_) while facilitates neurons in the inverted pathway (Inv_*L*_). The strongly responded Inv_*L*_ neurons drive downstream Dec_*R*_ decision neurons which in turn activate saccade neurons (in Sac_*R*_) and trigger an antisaccade. The model exhibits two types of errors, fast and slow, in antisaccade trials. **C** Fast errors are produced due to the subjects being unable to withhold a saccade against the direct target signal input. The decisions were not yet reached in the decision layer in the remapping module when the erroneous saccades were triggered by the action-selection module. **D** Slow errors originate from wrong decisions made in the decision layer (middle panel). In these trials the subjects were able to withhold a saccade initially against the strong input from the target signal. But the subsequently arrived signal from the decision module carries the wrong information.

Next, we performed full model simulations and examined how the circuit works to produce prosaccades, antisaccades and erroneous responses. In a prosaccade trial (Fig 4A), due to the weak level of the gaze-holding control, the activity of saccade neurons (Sac_*L*_) in the action-selection module rose quickly in response to the visual input from the left target. At the same time, the left visual input strongly activated the direct pathway (Dir_*L*_ to Dec_*L*_) which in turn projected to the same saccade neurons. As a result, the saccade neurons in Sac_*L*_ received strong input and quickly triggered a prosaccade.

In an antisaccade trial (Fig 4B), the strong level of the gaze-holding control temporally suppressed the responses of the saccade neurons Sac_*L*_ & Sac_*R*_ to any input including the visual input from the left target. In the meaning time, the remapping control activated Inv_*L*_ neurons while suppressed Dir_*L*_ neurons. As a result, the downstream decision neurons received a stronger input in Dec_*R*_ than in Dec_*L*_. The neurons in Dec_*R*_ won the competition against Dec_*L*_ and activated the downstream saccade neurons (Sac_*R*_) which triggered an antisaccade to the right.

Based on the simulated neural activities, we suggest that the SEF neurons that involve in the direct or inverted pathways can be identified by comparing their related strength of visual responses during prosaccade and antisaccade trails (as depicted in Fig 4A and 4B)

### Fast errors and slow errors

When modelling a cognitive function, it is insightful to study how the nervous system makes errors in addition to how it makes correct responses. Indeed, we found that the proposed model makes two types of erroneous responses in antisaccade: “slow error” and “fast error” (Fig 4C and 4D). In some antisaccade trials, the system exhibited a weak gaze-holding control which could not efficiently suppress the response of the saccade neurons (Sac_*L*_) to the left target. Therefore, a quick prosaccade was triggered even before the upstream decision layer (Dec_*R*_ and Dec_*L*_) reached any decision (Fig 4C). In other antisaccade trials, due to the stochastic nature of the neural competition, activity of Dec_*L*_ neurons ramped up against Dec_*R*_ and an incorrect decision was made. In this case, even if the system exhibited a strong gaze-holding control and successfully suppressed the response of Sac_*L*_ neurons to the onset of the left target, the wrong decision made in the upstream decision layer could still produce an erroneous response. The reaction times in this type of error trials were comparable to those of correct antisaccade trials because in both case the system went through the neural competition in the decision layer (Fig 4D). Since the model we used for the remapping module was originally used for near-threshold perceptual discrimination [35], this result suggests that a common mechanism for near-threshold decision-making in perceptual discrimination and conflict resolution between competing automatic and voluntary actions in behavioral control. The model further suggests that erroneous saccades with slow or fast reaction times are due to different mechanisms as revealed by the activity of neurons in the decision layer (Fig 4C and 4D), which presumably corresponds to the movement neurons in SEF.

**Fig 5:**
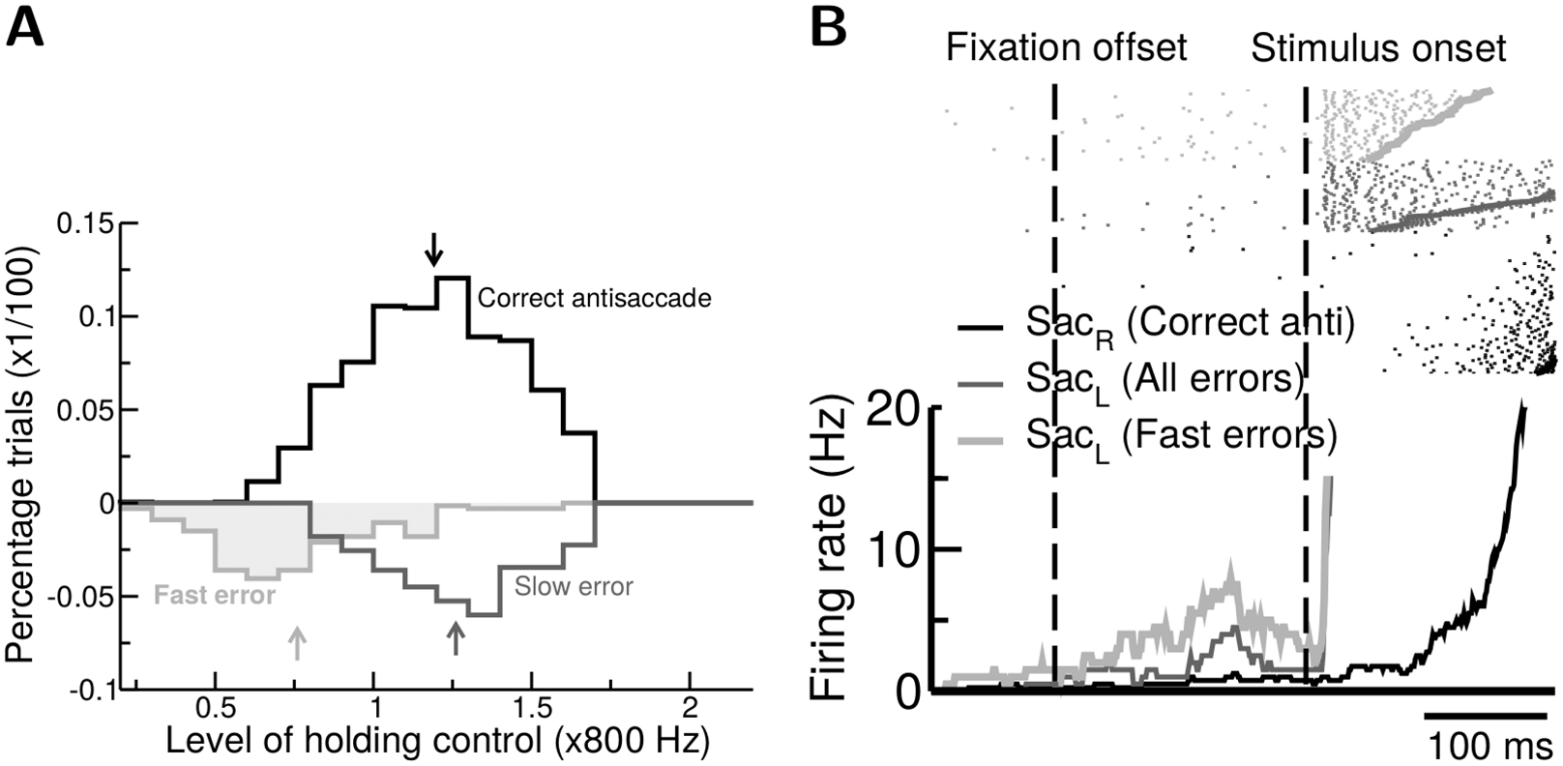
Fast errors in simulated antisaccade result from weak gaze-holding controls in the action-selection module. **A.** Distributions of the strength of gaze-holding control in three types of antisaccade responses: correct (black), fast errors (light grey) and slow errors (dark grey). Arrows indicate the mean of the corresponding distributions. Fast errors are significantly associated with weaker control levels. **B.** Spike rasters (top, thick lines denote saccade onsets) and trial averaged firing rate (bottom) from a simulated build-up neuron recorded in antisaccade trials. Trials with erroneous responses (dark grey) exhibit elevated activity prior to the stimulus onset comparing to the activity in correct antisaccade trials (black). The trend becomes more significant if we only select trials with fast errors (light grey). All activity shown here were recorded from the simulated Gap task.

We next verified that in the model the level of gaze-holding control indeed affected the types of error the system made. We first defined the “fast error” as the erroneous saccade with a reaction time shorter than that of all correct antisaccades (178 ms in the case of the gap paradigm demonstrated here) while the “slow error” are the remaining erroneous saccades with longer reaction times. We found that the trials with fast errors tended to have a weaker gaze-holding control (Fig 5A), whereas slow errors had comparable levels of gaze-holding control with that of correct antisaccade trials, indicating that the slow errors were produced due to a mechanism (neural competition in the upstream decision layer) other than a weak gaze-holding control. The low level of gaze-holding control results in a high level of the saccade neuron firing rate during the gap period (Fig 5B). In consequence, an erroneous saccade is more likely to be triggered immediately after the target onset. Therefore, the model predicts a negative correlation between the level of top-down control and the saccade neuron activity during the gap period for fast errors in antisaccade trials.

### Diversity in neuronal activities

**Fig 6:**
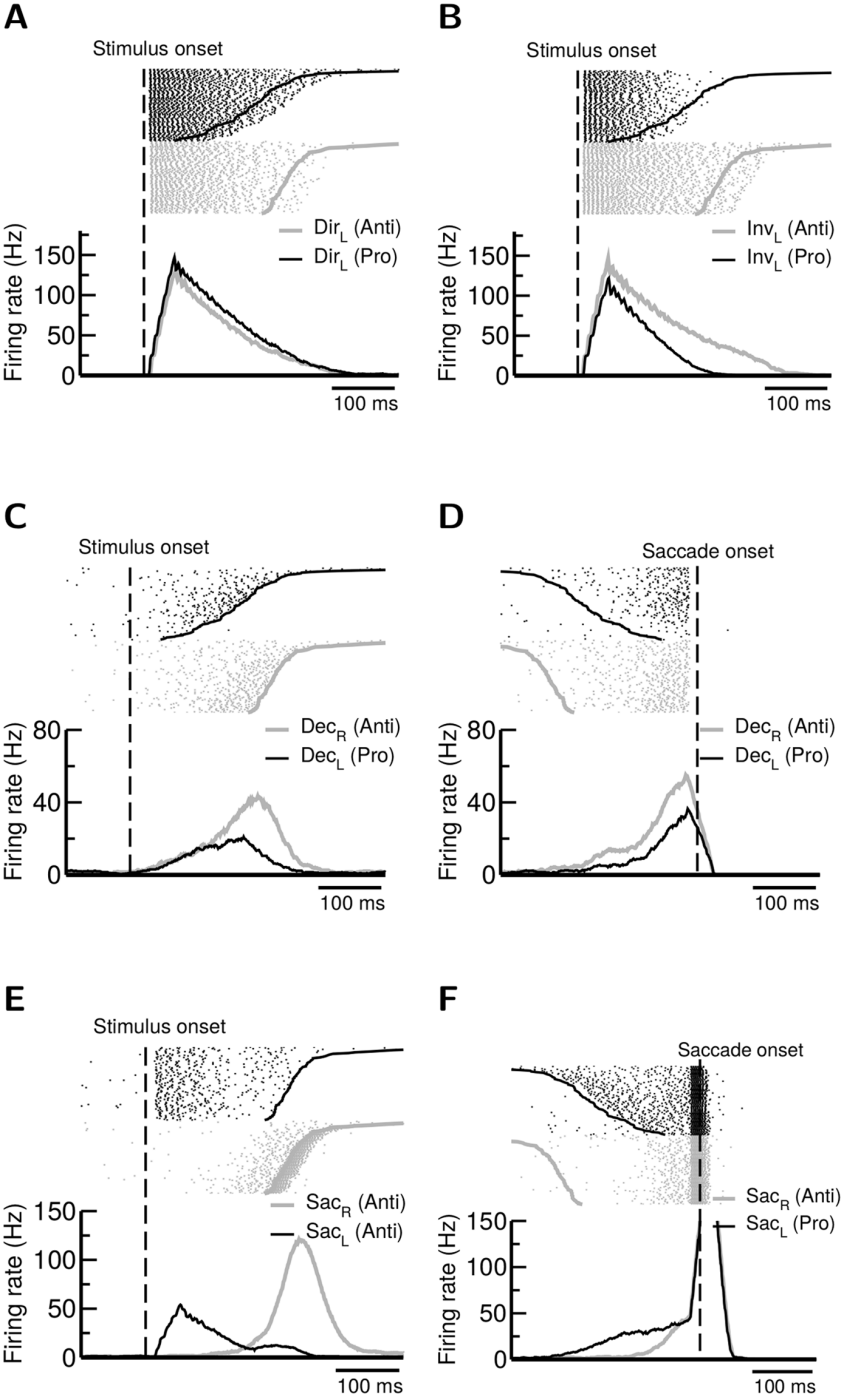
The model reproduces the observed diversity in the neuronal responses between prosaccade and antisaccade across brain regions. In all panels the target stimulus was presented on the left. Each panel displays spike rasters (top) and trial-averaged firing rates (bottom) from sample neurons. In the visual layer of the remapping module, depending on the type of recorded visual neurons we observe **A,** stronger prosaccade than antisaccade responses (Dir neurons) or **B,** stronger antisaccade than prosaccade responses (Inv neurons). **C & D.** Most movement neurons in the remapping module exhibit stronger antisaccade responses than prosaccade responses (**C**, align to the stimulus onset. **D,** align to the saccade onset). **E,** In the action-selection module, saccade neurons exhibit two waves of activity during antisaccade. Neurons receiving the direct visual stimulus develop a fast but weak response which is followed by a strong movement response on the correct side (right). **F,** if we compare the same neurons (Sac_*R*_ and Sac_*L*_) between prosaccade and antisaccade, the neuronal responses in the prosaccade trials are stronger than those of the antisaccade trials prior to the saccade onset. All activity shown here were recorded from the simulated Gap task. Activity exhibited in the NoGap or Overlap tasks is qualitatively similar.

The model qualitatively produced diverse neuronal activities observed in various brain regions [41–44]. We first examine the model-produced neuronal activities here and compare the model with observations in a later section. Neurons in the visual layer exhibited quick firing activity following target onset and we found that these neurons gave rise to stronger responses to the visual input in prosaccade trials than in antisaccade trials if we recorded from the direct map (Dir) (Fig 6A). In contrast, neurons in the same layer exhibited stronger activity in antisaccade trials than in prosaccade trials if recorded from the inverted map (Inv)(Fig 6B). This activity is consistent with several empirical studies in which some visual neurons in SEF were found to exhibit a stronger response to the visual target in antisaccade than in prosaccade, while a few other visual neurons in SEF exhibited an opposite trend [42, 46](S1 Fig).

Neurons in the decision layer can be viewed as movement neurons because they develop strong activity toward the onset of the motor responses. Interestingly, if we recorded neurons from the side that corresponded to the correct response (Dec_*R*_ for antisaccade and Dec_*L*_ for prosaccade), correct antisaccade produced stronger responses than correct prosaccades did (Fig 6C and 6D). This is because in prosaccade trials the direct visual activation from Vis_*L*_ neurons to Sac_*L*_ neurons also contributed to the generation of saccades. Therefore a prosaccade could often be triggered when the activity of neurons in Dec_*L*_ was still weak. Interestingly, it was reported that most movement neurons in SEF exhibited stronger activity in antisaccade than in prosaccade [42, 46]. This is consistent with the response of the neurons in the decision layer we report here (S2 Fig).

In the action-selection module, we observed that the saccade neurons (Sac) developed two waves of activities in antisaccade trials. The neurons (Sac_*L*_) that received the visual input exhibited a moderate response immediately following the target onset but became suppressed due to the inhibition from the gaze-holding control. On the other hand, the neurons on the opposite side (Sac_*R*_) started to develop a strong activity upon the arrival of the antisaccade signal from the upstream decision layer (Fig 6E). Such two waves of activity could be identified in several empirical observations of neurons in FEF or SC in which neurons with the target in their response field showed a transient but strong response with an early onset, while a slightly weaker activity with a later onset was developed in neurons in the opposite side when a correct antisaccade was initiated [43, 44, 47, 48](S3 Fig). However, in the model the early response was weaker than the latter response. Interestingly, in a study using visual search task combined with prosaccade and antisaccade [44], two types of neurons were observed in FEF. Type I neurons responded to the target (singleton) and the saccade endpoint with a “two-wave” activity in antisaccade-like trials. This type of neurons are similar to the build-up neurons (BUN) in the Sac populations in our model. Type II neurons only responded to the saccade endpoint with a monotonic ramping activity which resembled the activity of neurons in the decision layer in our model. Therefore, the mappings between the model components and the brain regions may not be one-to-one but multiple-to-multiple, dependent on the specific task being investigated.

Futhermore, by comparing saccade neurons that triggered correct prosaccade (Sac_*L*_) and correct antisaccade (Sac_*R*_), we found that prosaccades produced stronger responses than antisaccades did before the target onset (Fig 6F). This trend is consistent with several earlier studies which reported that saccade neurons in FEF and SC exhibited stronger population activity in prosaccade than in antisaccade before the saccade onset [41, 43](S3 Fig). However, around and after the saccade onset, observed SC neurons still displayed stronger prosaccade than antisaccade activity. The observation was not reproduced in our model.

### Behavior performance

**Fig 7:**
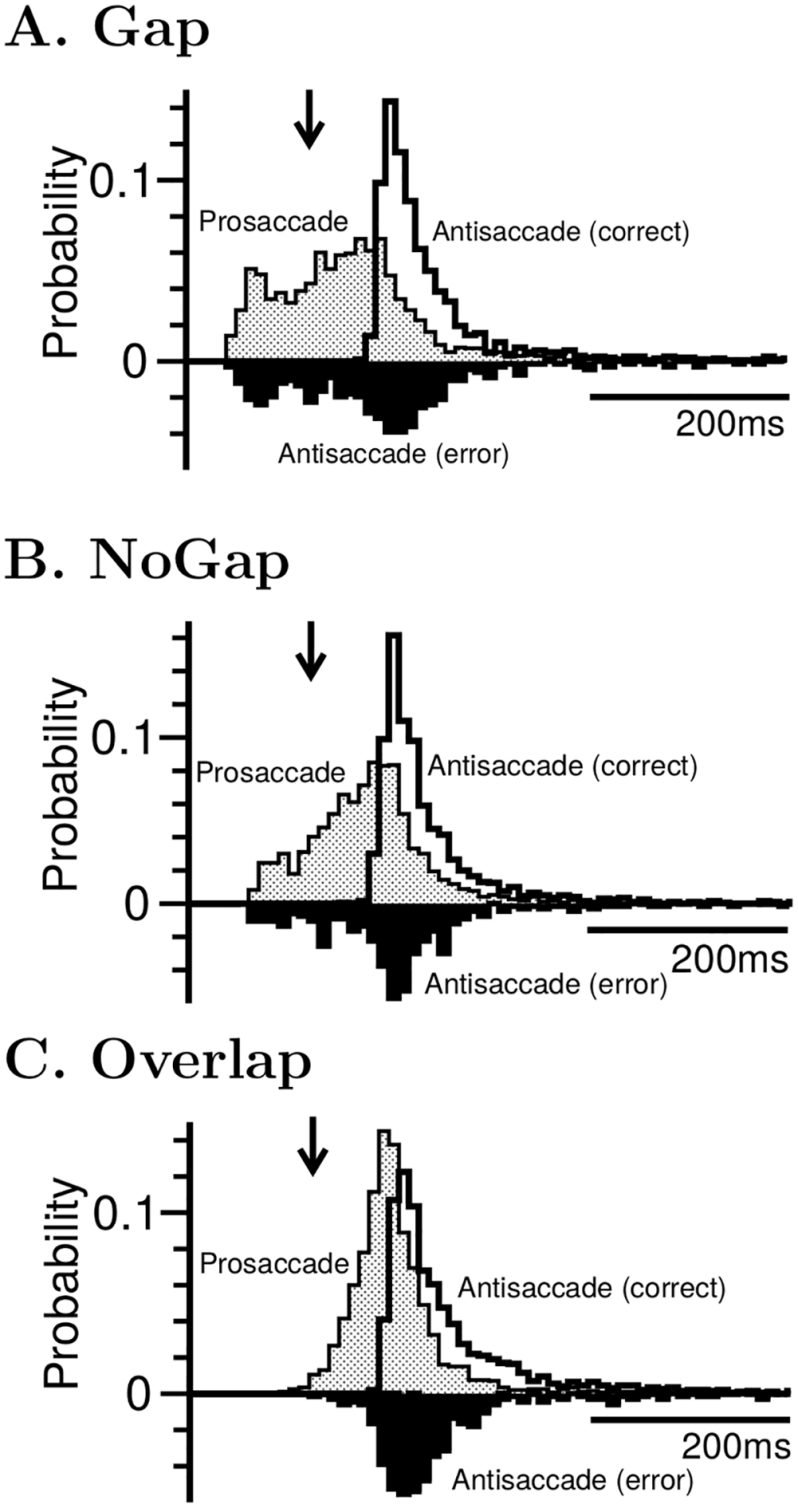
The model produces reaction time distributions in consistent with those observed in experiments. Here we plot the distributions for antisaccade (correct: white, error: black) and prosaccade (shaded) in the **A** Gap, **B** NoGap and **C** Overlap tasks. In the Gap task, the offset of the fixation signal prior to the onset of the target weakens the subject’s ability to withhold the gaze, which is reflected in the large number of express saccades (RT < 125ms, indicated by arrows) both in the prosaccade and in the erroneous responses in antisaccade trials. This trend is less significant in the NoGap task and disappears in the Overlap task. Due to the relatively small percentage of erroneous responses, the heights of their distributions have been magnified by three-fold for easy viewing.

Next, we examined the reaction time distributions produced by the model in the gap, no-gap and overlap paradigms (Fig 7). The mean reaction time of correct antisaccade was longer than that of correct prosaccade in all three paradigms. The mean reaction time of failed antisaccade trials (erroneous prosaccade) were shorter than that of correct ones. Notably, reaction times of the erroneous antisaccade covered a broad range with some erroneous responses as fast as the fastest prosaccades and others as slow as the slowest antisaccades. The Gap paradigm produced the highest percentage of fast errors in antisaccade among the three paradigm while the Overlap produced the least. Furthermore, the reaction times of prosaccade and erroneous antisaccade also exhibited bimodal distributions. This bimodal feature was most significantly in the gap paradigm, becomes less apparently in no-gap paradigm and disappeared in overlap paradigm. All of these features were observed in earlier empirical studies of prosaccade and antisaccade using the Gap and Overlap paradigms [41, 45] (S4 Fig). However, the detailed shape of reaction time distribution is strongly subject-dependent and the bimodality may not be observed in all subjects.

**Fig 8:**
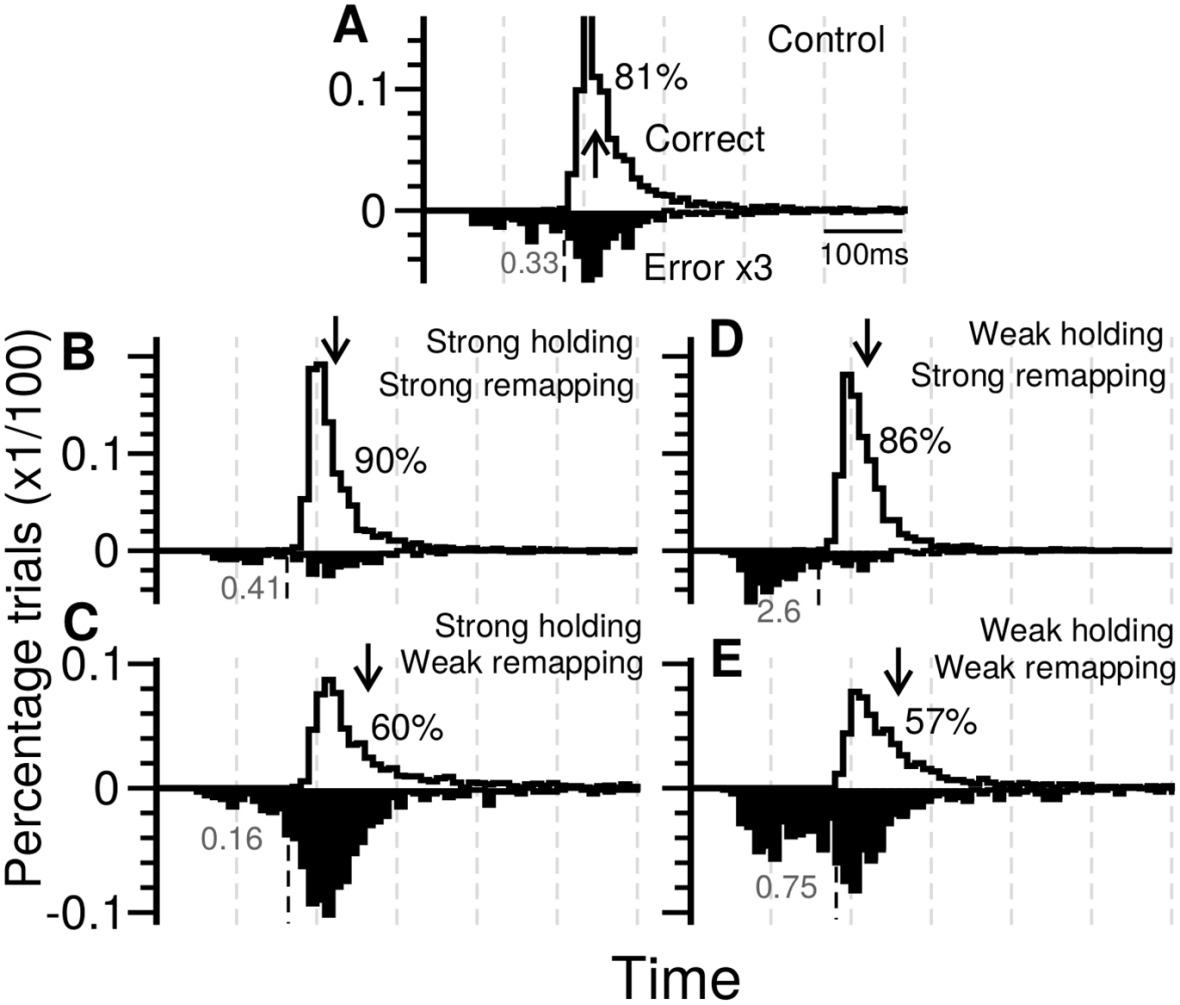
Gaze-holding control and remapping control both influence the behavioral outcome but with different effects. **A.** Reaction time distributions for the antisaccade trials with correct (white) and erroneous (black) responses in the Gap task. The distributions of erroneous responses in antisaccade have been magnified vertically by three-fold as in Fig 7. The black number to the right of the distribution of correct responses represents the percentage of correct trials. The grey number below the abscissa indicates the ratio between the numbers of fast and slow errors. **B-E,** same with **A** but with different strengths of gaze-holding control and remapping control. The strength of the remapping control affects the percentage correct, the mean reaction time and the ratio of fast/slow errors while the strength of the gaze-holding control mainly affects the ratio of fast/slow errors. Arrows indicate the mean RT of correct antisaccades in each panel.

It is interesting to see how the model predicts when the levels of the remapping control and/or the holding control change. To this end, we performed simulations under the following four conditions of control levels: 1) strong holding / strong remapping, 2) strong holding / weak remapping, 3) weak holding / strong remapping and 4) weak holding / weak remapping. The strong and weak remapping controls were simulated using levels that are 20% more or 20% less than the normal level used in Fig 7B, respectively. The strong gaze-holding control was also modelled as 20% more than the normal level while the weak gaze-holding control is simply modelled as the level used in prosaccade trials. We examined the reaction time distribution and the error rate of antisaccades and found that the level of the remapping control mainly affected the percentage correct and the mean reaction time of correct antisaccade (Fig 8). A strong or a weak remapping control led to 90% or 60% of correct antisaccade trials in the case of strong gaze-holding control. The mean reaction time of correct antisaccades was ∼ 225 ms for the strong remapping control and ∼ 264 ms for the weak remapping control. On the other hand, the level of the gaze-holding control significantly affected the percentage correct but not the mean response time. The relative numbers of fast errors versus slow errors were affected by both control types, but in opposite ways (Fig 8). A strong gaze-holding control resulted in lower ratio of fast errors because of the suppression of automatic responses while a strong remapping control increased the ratio of the fast errors due to the fewer slow errors made by the decision layer. This model predicited association between the ratio of fast/slow errors and the deficit of top-down control may be used for clinical assessment (see Discussion).

## Discussion

In the present study we constructed a spiking neural network model which demonstrates how the brain selects between competing automatic and voluntary responses in the antisaccade task. Our model distinguishes previous models [28–33] in three basic respects. First, a near-threshold decision circuit makes a decision on the saccade direction, whereas the motor output is determined in a separate, action-selection module. Second, competition between the automatic response and voluntary response occur in two stages in more than one brain regions through different neural pathways. Third, top-down control has two components; the stop, or action-holding, control does not race against the go process, but exhibits proactive inhibition through tonic activation of fixation neurons.

Each of the three assumptions play different roles in reproducing the diverse behavioral and neuronal activity observed in the antisaccade task. The decision circuit reproduces the basic behavioral trait of antisaccade, which is characterized by the slow and less accurate responses. The two-stage competition further produces express saccades in prosaccade trials, fast errors in antisaccade trials and diverse neuronal activity observed in different brain regions (see the detailed discussion below). The two-component top-down control provides predictions of the model by showing that the manipulation of the individual components can change the error rate and the ratio between the fast and slow errors in the antisaccade trials (see the detailed discussion below).

The two-stage competition scheme is essential in reproducing behavioral and neuronal observations in the antisaccade task. In the scheme (1) The target signal coming down the direct visual input pathway competes with the voluntary signal under the modulation of gaze-holding control and (2) the target signal coming down the remapping pathway competes with the inverted saccade signal (produced by the remapping top-down control) in the decision layer with a mechanism resembles near-threshold decision-making. A successful antisaccade requires that the voluntary response (driven by the top-down controls) wins both competitions. On the other hand, erroneous prosaccades made in antisaccade trials can be caused by two distinct mechanisms. Errors with fast response times are produced due to a weak gaze-holding control. In this scenario, the direct visual stimulus from the target activates the saccade neurons in the action-selection module before the remapping module is able to generate the signal that inverts the saccade direction. Errors with slower response times are produced due to the failure of the remapping module in inverting the saccade direction even when the gaze-holding control successfully suppresses the response of the action-selection module to the direct visual input from the target. This approach utilizes neural competition in the attractor network (implemented in the remapping module) in which a smaller difference in the inputs to the two competing neural pools results in a smaller percentage correct and a longer mean reaction. It has been shown that the erroneous responses in such a network have a similar mean reaction time to that of correct responses [35]. Hence, it is suitable to account for slow errors with long reaction times comparable to those of correct antisaccade. It remains to see whether this model conclusion is generally valid, for instance by examining in future work whether slow errors are present in Stroop task [1, 49, 50] and other behavioral paradigms that engage inhibitory control. The two competition pathways also explain why in the superior colliculus (and in the frontal eye field) the prosaccade responses are stronger than antisaccade responses while in the supplementary eye field the neural responses are diverse (some are stronger in prosaccades and others are stronger in antisaccades) and the diversity distributed differently across visual and movement neurons.

Our modelling result implies that when using the antisaccade task as a diagnostic tool, in addition to measuring the reaction times and the percentage of correct antisaccades, assessing the erroneous responses including the ratio between the fast and slow errors may also provide valuable information. For example, when a subject has an moderate impairment in the holding control, it does not change the mean reaction time for the correct antisaccade and only slightly reduces the percentage correct. However, if we measure the percentage of the fast errors, the impact of the impaired holding control becomes very significant (Fig 8).

A new insight from the model is that the top-down control consists of two components: remapping and action holding controls. The holding control exhibits an inhibitory effect on the action selection module. This inhibition is thought to be exhibited by the prefrontal cortex [4–9]. However, several recently studies on DLPFC deactivation demonstrated more complex effects, including excitatory influence of DLPFC on the antisaccade tasks [11, 48]. The observations implied that, if there is inhibitory top-down control involved in the antisaccade task, it may be carried out by brain regions other than DLFPC. In the proposed model, the gaze holding and the remapping controls exhibit inhibitory and excitatory effects, respectively, over the action-selection module. Therefore, these two controls may not originate from a single prefrontal region, but rather from a distributed network across multiple brain regions that extend beyond DLPFC [10].

A recently large-scale neural network model with a similar design of multiple control mechanisms has been independently developed and shown to be able to simulate various inhibitory control tasks including antisaccade [31]. The model consists of two main control pathways: 1) from the conflict detecting anterior cingulate cortex to the hyperdirect pathway in basal ganglia and 2) from the rule-dependent dorsolateral prefrontal cortex (DLPFC) to the indirect pathway in basal ganglia and to frontal eye fields. Although the first mechanism is functionally similar to our top-down inhibition on the action selection module, the latter one is different from ours. In our model, we have a top-down re-mapping signal that facilitates the inverted pathway in the antisaccade trials. As a result, the competition (or the “conflict”) between the direct and inverted pathways increases and the resulting mean reaction time in the antisaccade trials is slower than that of prosaccade trials. In Wiecki & Frank 2013, the larger antisaccade response times are mainly due to the slow responses of DLPFC because of the larger membrane time constant but not the result of the stronger competition. Unlike in the present model which produces two types of errors, in their model the error in antisaccade is mainly due to failing to suppress prosaccades and therefore the response times are faster than those of the correct antisaccade trials.

The present work focuses on neural processes that resolve conflict in a trial and that lead to diverse erroneous responses in antisaccade. We do not model the slow neural processes that exhibit the influence across trials. For example, erroneous antisaccades may be frequently made in prosaccade trials when preceded by a block of consecutive antisaccade trials [51]. This type of trial-history effect can be reproduced in our model by introducing a slow and history-dependent component in the remapping control (as the task-set inertia) and/or in the gaze-holding control (as the persistent response-system inhibition) [52]. We also do not model trial-to-trial adaptation [53] which requires conflict monitoring in ACC [54]. It is interesting to incorporate a history-dependent modulation in the control system and/or an ACC module with a learning process in an extension of our model in the future. Another interesting open question for future research is the rule representation itself. Although there are some physiological studies of it [55], we are still not clear about the rule circuit (presumably in the prefrontal cortex) and how it actually implements the control signal.

One may argue that the spiking neuron model is not necessary for our circuit model because the top-down control and the competition pathways mainly work at the circuit level, at which the detailed neuron dynamics does not seem to be important. However, our neuron model is not just “spiking”, but also endowed with conductance-based synapses which captures temporal dynamics of synapses. In particular, the slow reverberatory excitation mediated by the NMDA receptors is crucial for the winner-take-all decision circuit [35] used in the proposed model. Indeed, recent studies have demonstrated the rule of NMDA receptors in antisaccade [56, 57]. Our biologically-based model allows us to further investigate how manipulation of specific receptors may influence the performance of antisaccade in future studies.

In the present study we do not explicitly include basal ganglia in the model. Basal ganglia controls eye movements by inhibiting or disinhibiting neurons in SC and hence may participate in the antisaccadic eye movement [58–62] (however, see Condy et al. 2004 [63]). Indeed, in Wiecki & Frank model [31], the authors included basal ganglia which plays a central role in action control. So, why can our model, without including the basal ganglia, still reproduce a broad range of behavioral and neuronal activities that observed empirically? From the functional point of view, the inhibitory control in the basal ganglia is replaced by the holding control and fixation neurons in the action selection module in our model. Furthermore, comparing to the fixation neurons which perform functions specifically for gaze holding, the inhibitory pathways (indirect and hyperdirect) in basal ganglia is multi-functional and is more associated with higher brain functions including inhibition of planned responses, enhancement of action precision, avoidance of aversive stimuli, etc [58, 59, 64–66]. Although a number of studies discovered differential responses of striatal neurons to prosaccade and antisaccade [60, 61], a brain lesion study did not found a significant impact of the basal ganglia lesion to the performance of antisaccade [63]. Moreover, studies on psychiatric disorders that associated with basal ganglia abnormality, e. g. Parkinson’s and Huntington’s diseases, found mixed results in their effects on antisaccade (see [17] for an extensive review). In addition to basal ganglia, other brain regions, such as inferior parietal lobule, middle occipital gyrus and cuneus, have also been suggested to be involved in antisaccade [67]. Further studies on precise lesions or deactivation of related brain regions are necessary to reveal actual contributions of these regions to the performance of antisaccade. Our model can be viewed as providing a minimum or a principle circuit that includes only major functional modules necessary for performing antisaccade. Some of the elements in the model may be actually implemented in multiple locations in the brain as redundancy.

In conclusion, the proposed model suggests a two-stage competition mechanism for how the brain selects between the automatic and voluntary responses in the antisaccade task. In this mechanism, the voluntary responses driven by top-down control competes against the automatic responses in two different pathways. Failing in either of the pathways results in erroneous responses. The proposed model is able to reproduce a wide range of neuronal and behavioral features observed in various studies and provides insights into how competing responses are selected at the neural circuit level and why the subjects make errors. This work demonstrates that a common mechanism, with a combination of NMDA receptor mediated slow reverberatory excitation and winner-take-all-competition, is at the core of both near-threshold perceptual decision-making and behavioral control.

## Materials and Methods

### Behavior task: Antisaccadic eye movement

We developed a model for resolving a conflict between automatic and voluntary responses. For the sake of concretness, we tested the model by simulating a pro- versus anti-saccade task (Fig 1C). In the beginning of a trial, the subject fixated at the spot, or the fixation signal, located at the center of the screen. The color of the fixation signal served as a cue indicating the type of the response (green for prosaccade and red for antisaccade). After a delay, a target appeared on the either side of the screen. In prosaccade trials, the subject had to make a saccade into the target as soon as it appears. In antisaccade trials, the subject had to make a saccade to the side that opposites to the target. In the present study we tested the model using three different paradigms of antisaccade: Overlap, Gap and NoGap (Fig 1D). This three paradigms were different in the timing of the fixation signal offset. In Gap task, the fixation signal turned off 200 ms before the onset of the target. In Overlap task, the fixation signal was not turned off during the entire course of the trial. In the NoGap task, the fixation signal was turned off at the same time with the target onset.

In the simulations the stimuli including the fixation signal and the target signal were modelled as excitatory inputs to the fixation neurons and visual neurons, respectively. The cue determines the levels of the top-down controls in the model. See the sections “The network model” and “The visual stimuli and top-down controls” below for details. A saccadic eye movement was triggered in the simulation if the population firing rate of one of two (right and left) burst-neuron populations in the action-selection module exceeded 100 Hz. The population firing rate was calculated using a 20 ms sliding time window. The reaction time was defined as the interval between the onset of the target and the saccade.

### Single neuron and synapse models

Single neuron and synapse models followed those used in previous studies [35, 36]. Briefly, each neuron in the circuit model was simulated using the leaky integrate-and-fire model with conductance-based synapses. The membrane potential *V*(*t*) for each neuron obeys the following equation:

$$C_{m}\frac{dV(t)}{dt}=-g_{L}\left(V\left(t\right)-V_{L}\right)-I_{syn}\left(t\right),$$

where *C*_*m*_ is the membrane capacitance, *g*_*L*_ is the leak conductance, *V*_*L*_ is the resting potential and *I*_*syn*_ is the total synaptic current. When the membrane potential *V*(*t*) of each neuron reaches a threshold *V*_*threshold*_ = −50 mV, a spike is emitted and *V*(*t*) is set to the reset potential *V*_*reset*_ = −55 mV for a refractory period *T*_*r*_ = 2 ms. For inhibitory neurons, we used the following parameters: *C*_*m*_ = 0.2 nF, *g*_*L*_ = 20 nS and *V*_*L*_ = −70 mV. For excitatory neurons, we used *C*_*m*_ = 0.5 nF, *g*_*L*_ = 25 nS and *V*_*L*_ = −70 mV.

The synaptic current *I*_*syn*_(*t*) includes external inputs from the outside of the modelled circuit (stimuli, top-down controls etc), background noise and internal input from other neurons in the modelled circuit. We modelled three types of synaptic receptors: AMPA, NMDA and GABA_A_. They are described by:

$$I_{syn}\left(t\right)=g_{\text{AMPA}}s_{\text{AMPA}}\left(t\right)\left(V\left(t\right)-V_{E}\right)+\frac{g_{\text{NMDA}}s_{\;\text{NMDA}}\left(t\right)\left(V\left(t\right)-V_{E}\right)}{1+\left[Mg^{2+}\right]e^{-0.062V(t)}/3.57}+g_{\;\text{GABA}}s_{\text{GABA}}\left(t\right)\left(V\left(t\right)-V_{I}\right),$$

where *V*_*E*_ (= 0) and *V*_*I*_ (= −70 mV) are the reversal potentials, [Mg^2+^] (= 1.0 mM) is the extracellular magnesium concentration, *g* is the synaptic efficacy and *s* is the gating variable. Subscripts in *g* and *s* denote the receptor type. The gating variable obeys

$$\frac{ds(t)}{dt}=\sum_{k}\delta \left(t-t^{k}\right)-\frac{s}{\tau }$$

for AMPA and GABA_*A*_ receptors and

$$\frac{ds(t)}{dt}=\alpha \left(1-s\left(t\right)\right)\sum_{k}\delta \left(t-t^{k}\right)-\frac{s}{\tau }$$

for NMDA receptors with *α* = 0.63. The decay constant *τ* was 2 ms, 5 ms and 100 ms for AMPA, GABA_A_ and NMDA receptors, respectively; *δ*(*t* − *t*^*k*^) is the delta function and *t*^*k*^ is the time of the *k*th presynaptic spike.

We implemented short-term facilitation (STF) in several synaptic connections in the action-selection module (indicated by * in Table 1). The gating variable *s* was multiplied by the STF factor *F*, which obeys the following dynamics [68]:

$$\frac{dF}{dt}=\alpha_{F}\left(1-F\right)\sum_{k}\delta \left(t-t_{k}\right)-F/\tau_{F},$$

where the dimensionless factor *α*_*F*_ was 0.15 and the decay constant *τ*_*F*_ was 1000 ms.

### The network model

Several brain regions have been found to play roles in antisaccadic eye movement. They include DLPFC [23–25, 69, 70], FEF [26, 43, 44, 47, 71], SEF [24, 42, 46, 71], SC [11, 41, 48], ACC [24, 71, 72], parietal cortex [67, 71, 73–75] and basal ganglia [59–62] (but see [63]). Some of the brain regions, such as frontal eye field and superior colliculus, exhibit similar neuronal responses during the antisaccade task, possibly reflecting a redundancy of neural representations. Based on this consideration, our goal is not to simulate activities in every correlated brain region, but to model the core neural processes that is sufficient to reproduce the diverse neuronal and behavioral responses.

The network model consisted of two major modules: the action-selection module (Fig 2A) and the remapping module (Fig 2B). The visual target signal reached the action-selection module which produced saccadic eye movements through two pathways: 1) a direct projection from the visual neurons Vis to the action-selection module and 2) a remapping pathway that went through the remapping module before reaching the action-selection module. The function of the remapping module was to map the visual signal input to the desired saccade direction, based on a previously proposed attractor network model of perceptual decision [35], and produces neural commends that drove the downstream action-selection module. The model was endowed with a cue-dependent top-down control component which modulated the action selection and the remapping modules in order to produce prosaccade or antisaccade. The functions of each module and their relationship are illustrated and analysed in Results. The parameters of synaptic connections of each neural populations are listed in Tables 1–3. All synaptic connections in the model were all-to-all, i.e. every neuron in the source population connected to every neuron in the target population.

**Table 2:** Synaptic conductance (nS) of connections in the remapping module. Letters preceding each number indicate the type of receptor. N: NMDA, A: AMPA, G: GABA

Source population(number of neurons)	Target population			
	Exc_*bg*_	Dec_*L*_	Dec_*R*_	I
Dir_*L*_ (240)		A 0.012		
Dir_*R*_ (240)			A 0.012	
Inv_*L*_ (240)			A 0.012	
Inv_*R*_ (240)		A 0.012		
Exc_*bg*_ (1120)	A 0.0719	A 0.0618	A 0.0618	A 0.0567
	N 0.160	N 0.1371	N 0.1371	N 0.126
Dec_*L*_ (240)	A 0.0719	A 0.130	A 0.0618	A 0.0567
	N 0.160	N 0.287	N 0.137	N 0.126
Dec_*R*_ (240)	A 0.0719	A 0.0618	A 0.130	A 0.0567
	N 0.160	N 0.137	N 0.287	N 0.126
I (400)	G 1.40	G 1.40	G 1.40	G 1.07

**Table 3:** Synaptic conductance (in nS) of connections between modules. Letters preceding each number indicate the type of receptor. N: NMDA, A: AMPA, G: GABA.

Source population	Target population			
	To action-selection module			
	BN_*L*_	BUN_*R*_	BN_*R*_	BUN_*R*_
Dec_*L*_	A 0.11	A 0.11		
Dec_*R*_			A 0.11	A 0.11
	**From action-selection module**			
	Exc_*bg*_	Dec_*L*_	Dec_*R*_	I
BN_*L*_	A 0.25	A 0.25	A 0.25	A 0.5
BN_*R*_	A 0.25	A 0.25	A 0.25	A 0.5

### Visual stimuli and top-down control signals

The model was driven by four types of inputs: background noise, target signal, fixation signal and top-down controls, which were all modelled as synaptic input with Poisson statistics to related neural populations through AMPA mediated currents. The level of background noise and the target signal are the same in all trial types. The fixation signal is elicited by the fixation point, hence is different between the Overlap, NoGap and Gap trials. The top-down controls, which consist of the gaze-holding control and the sensorimotor remapping control, are used to initiate an antisaccade.

The background noise was used to maintain each neuron at a desired baseline activity. The level of the background noise input to each neural population is listed in Table 4.

**Table 4:** Levels of the background noise for each neural populations in the model. Values are given in firing rate (x1000 Hz) / synaptic conductance (nS). All noise inputs are added to the populations as synaptic input with Poisson statistics through AMPA mediated currents.

Population	Vis	BN	BUN	I_0_	I_1,4_	I_2,3_	FN
Noise	2.4 / 0.3	1.28 / 0.15	1.28 / 0.5	1.28 / 2.0	1.28 / 2.0	1.28 / 1.6	1.6 / 2.0
Population	Dir	Inv	Dec	I	Exc_*bg*_		
Noise	5.44 / 0.3	2.0 / 0.3	2.4 / 2.1	2.4 / 1.62	2.4 / 2.1		

The target signal was sent to the visual neurons (Vis) in the action-selection module (Fig 2A) and to the visual neurons (Dir and Inv) in the remapping module (Fig 2B). To simulate the strong visual responses and the quick adaptation (at the hundred-millisecond time scale) commonly observed in many visually responded neurons, we modelled the firing rate of the target signal as

$$f\left(t\right)=\left(f_{\text{max}}-f_{\text{min}}\right)\exp\left(-t/\tau \right)+f_{\text{min}}\text{,}$$

where *t* = 0 corresponds to the onset of the target signal. At *t* = 0, the input firing rate jumps to *f*_max_ but decays exponentially to *f*_min_. In the study we set *f*_max_ = 28,000 Hz and *f*_min_ is 38.8% of the peak value *f*_max_. The decay time constant *τ* was 100 ms. The input conductance of the target signal was 0.3 nS for all populations (Vis, Dir and Inv) that receive the target signal input. Note that here and in the following, the input firing rate represents the total rate from a large number of upstream neurons.

The fixation signal drove fixation neurons in the action-selection module. The input firing rate was set to a constant value of 320 Hz with a synaptic conductance 2.0 nS. The fixation signal is elicited by the fixation point and is therefore turned off at the same time with the fixation point offset.

The top-down control depended on the rule signal (*C*_rule_) and consisted of two components: a gaze-holding control *C*_*h*_ and a sensorimotor remapping control *C*_rem_. When an antisaccade was required, *C*_rule_ = 1. Otherwise, *C*_rule_ = 0. In the beginning of each simulated trial, the rule signal *C*_rule_ is set to 0 or 1 according to the cue (the color of the fixation signal). Note that in the present study we focus on how conflict responses are resolved by the neural competition in multiple brain regions. The model was developed based on the assumption that the subjects already learned the association between the cue (the color of the fixation signal) and the task (prosaccade or antisaccade). Such associative learning can be realized by the mechanisms of flexible sensorimotor mapping proposed in other published studies (for example, the model proposed in Fusi et al 2007 [15]).

The gaze-holding control mimics a subject’s effort to withhold a gaze before the onset of the target and to suppress an express saccade triggered by the direct visual input. The gaze-holding control was modelled as a constant input to the fixation neurons (both FN_*L*_ and FN_*R*_) in the action-selection module with a synaptic conductance of 2.0 nS. The strength of the constant input varies from trial to trial and was given by

$$C_{h}=C_{h0}+kC_{\text{rule}}+\delta \text{,}$$

where *C*_*h*0_ (= 960Hz) is the mean strength of the gaze-holding control when a subject is actively fixating. The mean holding strength increased during an antisaccade trial because the subject tended to make more effort to suppress unwanted express saccades. This increase is described by the second term *kC*_rule_ where *k* = 140 Hz. The trial-to-trial variability was modelled in the third term. In each trial, the value of *δ* was determined by randomly drawing a number from a Gaussian distribution with a zero mean and a standard deviation of 240 Hz. To avoid drawing an extremely strong or a negative control level from the Gaussian distribution, we set an upper limit (400 Hz) and a lower limit (-960 Hz) for *δ*.

The gaze-holding control was turned on at the start of a trial and was turned off 150 ms after the onset of the target signal in all trial types. We noted that this offset latency, together with the trial-to-trial variability of the strength of gaze-holding control, were important in producing a desired reaction time distribution, especially in prosaccade trials. Therefore the related parameters were determined by matching the behavior outcome of the model to that of the typical experimental observations [41, 43].

The sensorimotor remapping control mimics a subject’s action to switch the response rule from prosaccade to antisaccade. In a prosaccade trial, no remapping control is needed and the Dir and Inv neurons only receive the target stimuli and the default background input as indicated in Table 3. In an antisaccade trial, the subject needs to suppress the direct map and facilitate the inverted map in the remapping module. Therefore, the remapping control was modelled as a reduced background input to the neurons (Dir) in the direct map and an increased background input to the neurons (Inv) in the inverted map. The amount of change in the background inputs is calculated using the following equations: $C_{\text{rem}}^{\text{Dir}}=k_{\text{Dir}}C_{\text{rule}}$ and $C_{\text{rem}}^{\text{Inv}}=k_{\text{Inv}}C_{\text{rule}}$, where *k*_Dir_ = − 1093Hz and *k*_Inv_ = 2000 Hz. $C_{\text{rem}}^{\text{Dir}}$ and $C_{\text{rem}}^{\text{Inv}}$ were then added to the background inputs indicated in Table 4. According to the equations, in prosaccade trials neurons in the direct map receive a larger background excitation than neurons in the inverted map whereas in antisaccade trials it is opposite.

The input firing rates (sensory and top-down control) described above seem to be arbitrary. However, they all fall into a physiologically reasonable range. Assuming that there are 240 neurons (same with the most populations in the model) in each upstream input neuron pool, the maximum sensory input of 28,000 Hz corresponds to 116.7 Hz per visual input neuron. Indeed, a large number of studies (Carandini & Ferster, 2000 [76] for example) on the mammalian visual cortex have reported such a strong response (> 100 Hz) to salient visual stimuli. Regarding the input associated with the gaze-holding or remapping controls, the mean strength of the controls in the antisaccade trials corresponds to a much smaller per-neuron firing rate (∼ 4-8 Hz). This is also a reasonable range considering that the top-down inputs are assumed to partially originate from the prefrontal cortex. Several studies have discovered the rule-dependent or cue-dependent neurons in the prefrontal cortex with a firing rate between 5Hz and 20 Hz, and rarely exceeding 50Hz [23].

In the model we did not manually add trial-to-trial variability to the remapping control as we did for the gaze-holding control. This is because the remapping module performed probabilistic decisions and already exhibited a high degree of trial-to-trial variability [35]. Adding more variability to the top-down remapping control, or equivalently, adding variability to the activity of Dir and Inv neurons did not help producing a better result. Once the subject applied the remapping control based on the rule signal, whether the response rule could be successfully switched was determined stochastically in the decision layer, where the trial-to-trial variability originated.

## Supporting Information

S1 Fig Comparison of the observed visual neurons in supplementary eye fields (SEF) and the neurons in the visual layer of the model. **A.** An observed visual neuron exhibited stronger responses in antisaccade than in prosaccade. **B.** Another observed visual neuron with an opposite trend. **C** Neurons in the inverted map of the model exhibit stronger visual responses in antisaccade than in prosaccade as the observed neuron shown in **A**. **D.** Neurons in the direct map of the model exhibit stronger visual responses in prosaccade than in antisaccade as the observed neuron shown in **B**. (**A** and **B** adapted from “Amador N, Schlag-Rey M, Schlag J. Primate antisaccade. II. supplementary eye field neuronal activity predicts correct performance. J Neurophysiol. 2004;91:1672–1689.” with permission. **C** and **D** adapted from Fig 6B and 6A, respectively).
(PDF)

S2 Fig Comparison of the observed movement neurons in supplementary eye fields (SEF) and the neurons in the decision layer of the model. **A.** Observed SEF neuron activity in the correct prosaccades (YYy) and correct antisaccades (NYy) in the preferred direction. (Adapted from “Amador N, Schlag-Rey M, Schlag J. Primate antisaccade. II. supplementary eye field neuronal activity predicts correct performance. J Neurophysiol. 2004;91:1672–1689.” with permission) **B.** Same as in **A** but with activity produced by the decision layer neurons in the model (adapted from Fig 6D).
(PDF)

S3 Fig Comparison of the observed superior colliculus (SC) activity and the saccade neuron activity in the model. **A.** Observed firing rates in the SC contralateral to the saccade direction in prosaccade trials (thick solid line) and antisaccade trials (dashed line). **B.** Same as in **A** but for activity produced by the model. **C.** Observed firing rates in the SC contralateral to the saccade direction in antisaccade trials (thick solid line) and contralateral to the stimulus in antisaccade trials (dashed line). **D** Same as in **D** but for activity produced by the model. (**A** and **C** adapted from “Everling S, Dorris MC, Klein RM, Munoz DP. Role of primate superior colliculus in preparation and execution of anti-saccades and pro-saccades. J Neurosci. 1999 April;19(7):2740–2754.” with permission. **B** and **D** adapted from Fig 6F and 6E, respectively).
(PDF)

S4 Fig Comparison of observed and model-produced reaction distribution. **A.** Observed reaction time distributions of prosaccade, antisaccade and erroneous prosaccade (shown as the black histograms below the abscissa) made in antisaccade trials in overlap and gap paradigms for two monkeys. (Adapted from “Everling S, Dorris MC, Klein RM, Munoz DP. Role of primate superior colliculus in preparation and execution of anti-saccades and pro-saccades. J Neurosci. 1999 April;19(7):2740–2754.” with permission.) **B.** Same as in **A**, but from the model simulations (adapted from Fig 7).
(PDF)
