# Supplementary figures and images for: Conflict Resolution as Near-Threshold Decision-Making: A Spiking Neural Circuit Model with Two-Stage Competition for Antisaccadic Task
Source: PLoS Comput Biol. 2016 Aug 23;12(8):e1005081. doi: 10.1371/journal.pcbi.1005081 (PMC4995026; doi:10.1371/journal.pcbi.1005081)

# S1 Figure

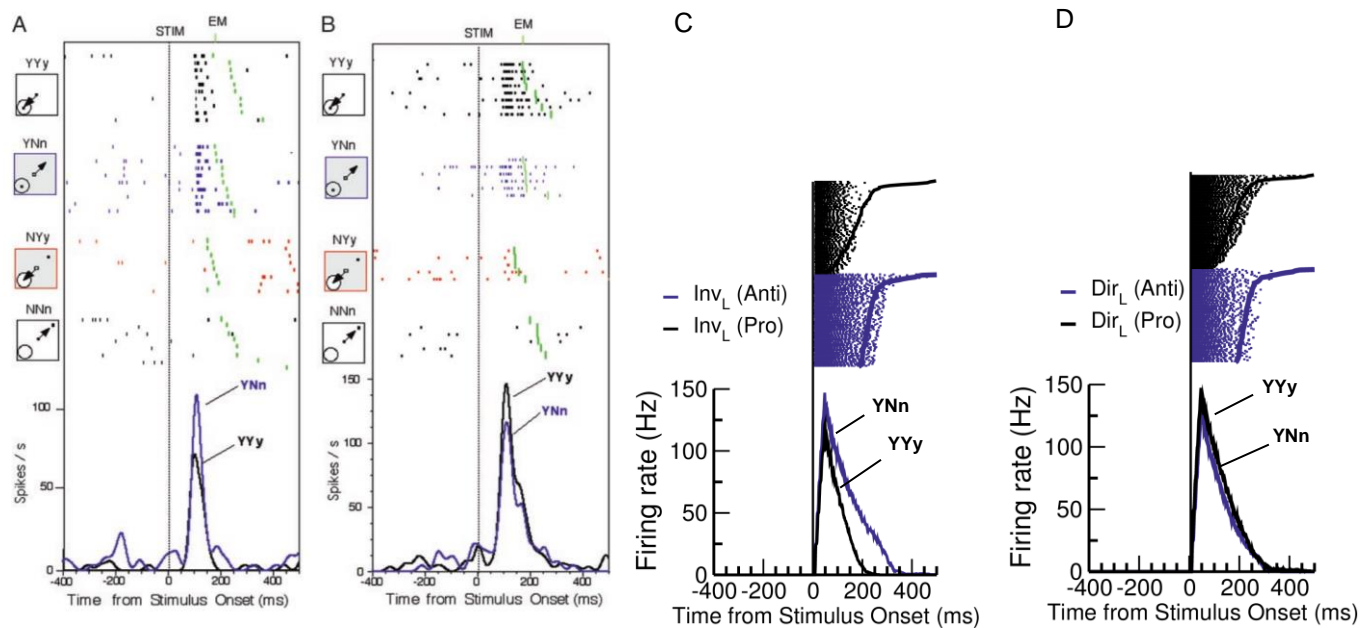

Supplement: S1 Fig — A. An observed visual neuron exhibited stronger responses in antisaccade than in prosaccade. B. Another observed visual neuron with an opposite trend. C Neurons in the inverted map of the model exhibit stronger visual responses in antisaccade than in prosaccade as the observed neuron shown in A. D. Neurons in the direct map of the model exhibit stronger visual responses in prosaccade than in antisaccade as the observed neuron shown in B. (A and B adapted from “Amador N, Schlag-Rey M, Schlag J. Primate antisaccade. II. supplementary eye field neuronal activity predicts correct performance. J Neurophysiol. 2004;91:1672–1689.” with permission. C and D adapted from Fig 6B and 6A, respectively). (PDF) [file pcbi.1005081.s001.pdf]

S2 figure

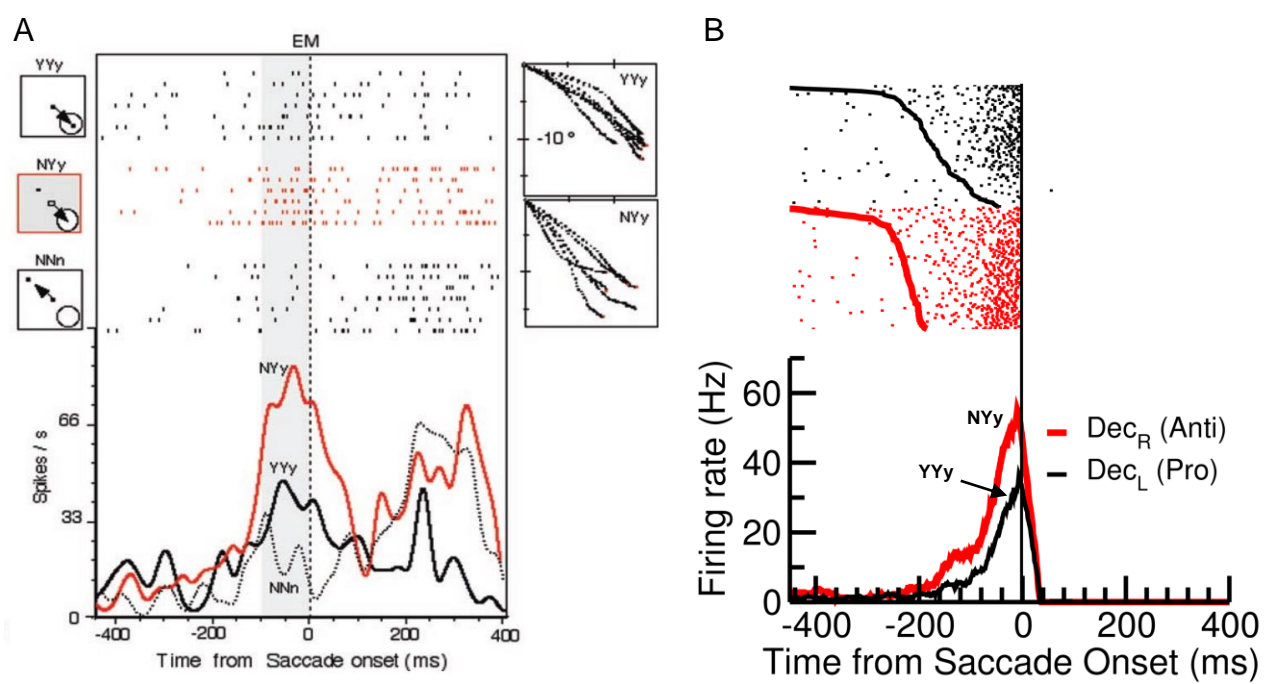

Supplement: S2 Fig — A. Observed SEF neuron activity in the correct prosaccades (YYy) and correct antisaccades (NYy) in the preferred direction. (Adapted from “Amador N, Schlag-Rey M, Schlag J. Primate antisaccade. II. supplementary eye field neuronal activity predicts correct performance. J Neurophysiol. 2004;91:1672–1689.” with permission) B. Same as in A but with activity produced by the decision layer neurons in the model (adapted from Fig 6D). (PDF) [file pcbi.1005081.s002.pdf]

S3 figure

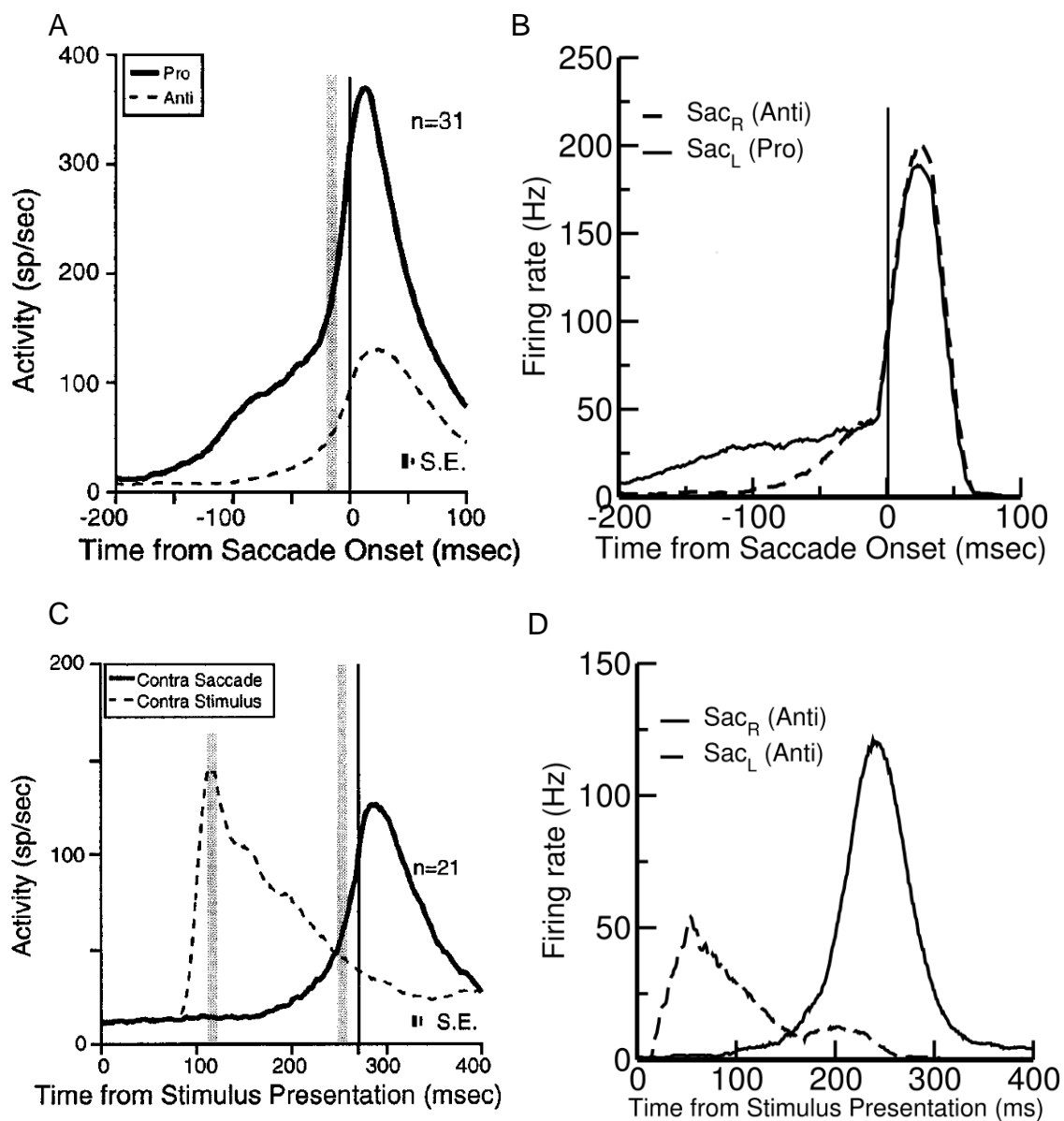

Supplement: S3 Fig — A. Observed firing rates in the SC contralateral to the saccade direction in prosaccade trials (thick solid line) and antisaccade trials (dashed line). B. Same as in A but for activity produced by the model. C. Observed firing rates in the SC contralateral to the saccade direction in antisaccade trials (thick solid line) and contralateral to the stimulus in antisaccade trials (dashed line). D Same as in D but for activity produced by the model. (A and C adapted from “Everling S, Dorris MC, Klein RM, Munoz DP. Role of primate superior colliculus in preparation and execution of anti-saccades and pro-saccades. J Neurosci. 1999 April;19(7):2740–2754.” with permission. B and D adapted from Fig 6F and 6E, respectively). (PDF) [file pcbi.1005081.s003.pdf]

S4 figure

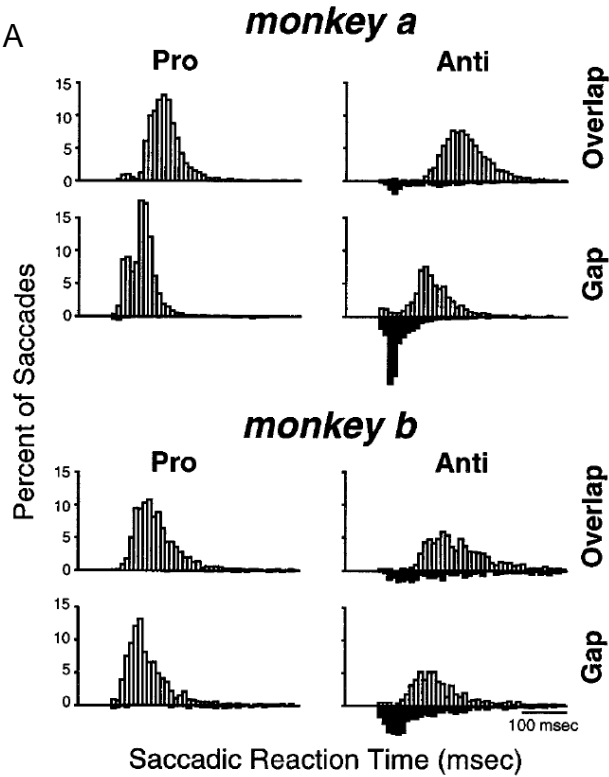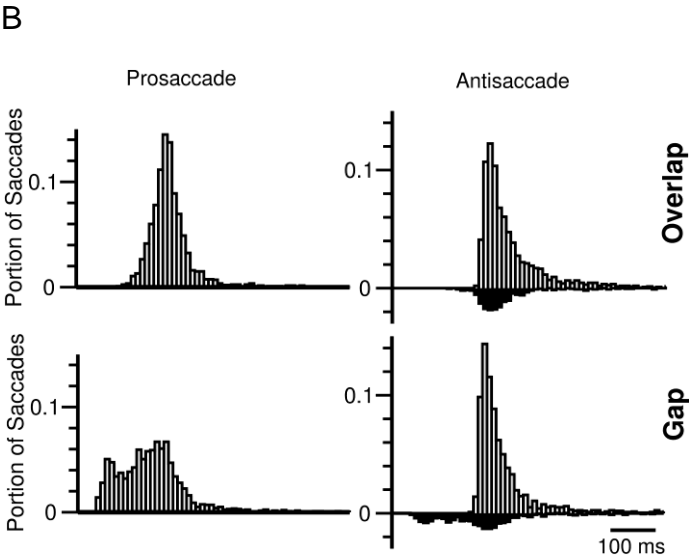

Supplement: S4 Fig — A. Observed reaction time distributions of prosaccade, antisaccade and erroneous prosaccade (shown as the black histograms below the abscissa) made in antisaccade trials in overlap and gap paradigms for two monkeys. (Adapted from “Everling S, Dorris MC, Klein RM, Munoz DP. Role of primate superior colliculus in preparation and execution of anti-saccades and pro-saccades. J Neurosci. 1999 April;19(7):2740–2754.” with permission.) B. Same as in A, but from the model simulations (adapted from Fig 7). (PDF) [file pcbi.1005081.s004.pdf]
